# Distributed Compressed Hyperspectral Sensing Imaging Based on Spectral Unmixing

**DOI:** 10.3390/s20082305

**Published:** 2020-04-17

**Authors:** Zhongliang Wang, Hua Xiao

**Affiliations:** 1 Department of Electric Engineering, Tongling University, Tongling 244061, Anhui, China; asdwzl@tlu.edu.cn; 2 Department of Mathematics and Computer, Tongling University, Tongling 244061, Anhui, China

**Keywords:** hyperspectral imagery, compressed sensing, distributed compressed sensing, linear mixing model, spectral unmixing

## Abstract

The huge volume of hyperspectral imagery demands enormous computational resources, storage memory, and bandwidth between the sensor and the ground stations. Compressed sensing theory has great potential to reduce the enormous cost of hyperspectral imagery by only collecting a few compressed measurements on the onboard imaging system. Inspired by distributed source coding, in this paper, a distributed compressed sensing framework of hyperspectral imagery is proposed. Similar to distributed compressed video sensing, spatial-spectral hyperspectral imagery is separated into key-band and compressed-sensing-band with different sampling rates during collecting data of proposed framework. However, unlike distributed compressed video sensing using side information for reconstruction, the widely used spectral unmixing method is employed for the recovery of hyperspectral imagery. First, endmembers are extracted from the compressed-sensing-band. Then, the endmembers of the key-band are predicted by interpolation method and abundance estimation is achieved by exploiting sparse penalty. Finally, the original hyperspectral imagery is recovered by linear mixing model. Extensive experimental results on multiple real hyperspectral datasets demonstrate that the proposed method can effectively recover the original data. The reconstruction peak signal-to-noise ratio of the proposed framework surpasses other state-of-the-art methods.

## 1. Introduction

Hyperspectral imagery (HSI) is different from conventional color images, and can collect tens or hundreds of spectrum samples for each image pixel. Therefore, HSI is usually used as a three-dimensional (3D) data cube with 2D spatial and 1D spectral variation [1]. This kind of data potential is useful in applications in the food safety, biomedical, forensic, and industrial fields [2]. However, with the increase in spatial and spectral resolution, the amount of data of HSI increases dramatically. This has motivated the application of compressed sensing (CS) [3] techniques for hyperspectral imaging.

CS is a mathematical framework for single-signal sensing and compression. CS theory has proved that sufficiently sparse signal can be accurately recovered from its compressed measurement by solving the quadratic programming [3]. Thus, only a few measurements need to be collected by CS technique to recover the original data. HSI can be transformed into sparse signals by many popular sparsification techniques such as orthogonal transformation-based methods [4], dictionary-based methods [5], or spectral unmixing [6,7].

As the double sparsity structure exists in both the spatial and spectral domains, a variety of sampling methods have been produced for HSI compressed sampling. First, spatial compressed sampling for conventional grayscale image can be applied directly to all spectral bands of HSI if the measurement matrix per channel is an independent random pattern, which is referred to as distributed CS (DCS) [8,9]. Second, the sparsity in spectral domains makes hyperspectral imagery easy to achieve with spectral compressed sampling. The most typical representative is compressive-projection principal component analysis (CPPCA) [10] and its derived algorithm [11,12,13,14]. However, CPPCA is only valid for the first few largest eigenvalues of hyperspectral imagery, but it is usually not true for the smaller eigenvalues, owing to the high degree of correlation among the spectral vectors [15]. Therefore, when the spectral sampling rate is very low, the CPPCA algorithm fails to recover original data. Previous works [9,16,17] on sampling mode have proposed compressed sampling hyperspectral imagery combining the structures of the spatial and spectral. Three-dimensional compressive sampling (3DCS) [16] constructed a generic 3D sparsity measure to exploit 3D piecewise smoothness and spectral low-rank property in hyperspectral imagery.

Inspired by distributed source coding (DSC) [18], the distributed compressive video sensing (DCVS) [19,20] framework was proposed for capturing and compressing video data simultaneously by integrating DSC and CS. DCVS divided the frames of a video sequence into key frames and non-key frames. Key frames are sent by conventional video lossless compression, and non-key frames are compressed sampled by common CS technology and transmitted to the decoder. Liu et al. [21] extended the DCVS framework to hyperspectral imagery. At the coding end, the compressed reference and non-reference band images and the prediction coefficients between them are collected. However, the calculation of prediction coefficient violates the original intention of CS sampling to be as simple as possible. In this paper, we divided hyperspectral imagery into key band images and CS band images, and then different sampling mode is applied to both types of images. With Kronecker product [9] transformation, the proposed compressed sampling method holds the same form as standard compressed sampling.

One of the important tasks of CS theory is how to recover the original data from a small amount of compressed data. The success of CS depends critically on the assumption that the underlying signals are sparse or compressible when represented on a suitable frame. Fortunately, hyperspectral imagery is highly correlated in both spatial and spectral domains and is thus compressible [15]. Many reconstruction algorithms are dedicated to development of sparse, total variation (TV), low rank, and other prior information for HIS [7,16,22,23,24]. However, this type of reconstruction algorithms performs convex optimization operations directly on the whole hyperspectral imagery. As there exists a huge amount of hyperspectral data, generally, the speed of such algorithms is slow.

Matrix decomposition is another kind of reconstruction approach for hyperspectral imagery. CPPCA [10] reconstructs an HSI data set using principal component analysis (PCA) at the decoder. The significant advantage for CPPCA is the transfer of computation from the on-board remote devices with limited computational resources to a ground working station. Although possessing excellent reconstruction quality and low computational complexity with high sampling rates, the reconstruction accuracy of CPPCA is low when the sampling rate lower than 0.2.

The linear mixing model (LMM) is one of the more simply and widely used hypotheses in hyperspectral imagery processing [2,25,26]. Hyperspectral imagery can be decomposed to endmember and abundance according to LMM. LMM-based compressed sensing reconstructing [6,15,27,28] for hyperspectral imagery demonstrated significant advantages in terms of both reconstruction quality and computational complexity. Spatio-spectral hybrid compressive sensing (SSHCS) [27] collects spatial and spectral compressed hyperspectral data, and recovers original hyperspectral imagery by the product of the endmembers extracted from the spatial compressed data and the corresponding abundance estimated from spectral compressed data. Spatial-spectral compressed reconstruction based on spectral unmixing (SSCR_SU) [28] extends SSHCS by alternately iterating endmembers and abundance. Spectral compressive acquisition (SpeCA) proposes a two-step measurement strategy operating on the spectral domain. One is the common spectral compressed sampling on per pixel, which is using to estimate abundance. The other is the spectral compressed sampling on some randomly chosen specific pixel, which can estimate endmembers by combining the estimated abundance.

In this paper, we propose a distributed compressed sampling and reconstruction framework for hyperspectral imagery. On the encoding side, we propose a distributed compressed sampling strategy similar to DCVS to collect hyperspectral data. The difference is that the side information of the key frame is used in DCVS reconstruction, which cannot be applied to the hyperspectral imagery in a low sampling rate environment with only a small number of key bands. Moreover, we recover hyperspectral data by linear spectral unmixing method on the decoding end. For brevity, we will call the proposed framework distributed compressed hyperspectral sensing (DCHS).

Specifically, the contribution of the paper has the following three aspects. First, distributed compressed sampling framework divides hyperspectral imagery into key band and CS band for separate acquisition, allowing endmembers and abundance to be independently estimated. Second, linear interpolation is employed to predict key band endmembers by the extracted CS band endmembers. Finally, an augmented Lagrangian minimization algorithm is designed to estimated abundance matrix under low sampling rate.

This paper is organized as follows. Section 2 proposes our DCHS framework. The endmembers predicting of key band and the augmented Lagrangian optimization algorithm for CS reconstruction by DCHS is described in Section 3. Section 4 presents the experimental results using three different datasets and discusses the quantitative and qualitative analysis. Finally, this study is concluded in Section 5.

## 2. Distributed Compressed Sampling Framework

The hyperspectral data of a single scene usually consists of several hundred images. Here, the matrix $X\in {\mathbb{R}}^{N\times L}$ describes the hyperspectral data of a particular scene. Each column of X represents a vectorized band image and each row denotes the spectrum of one pixel. The size L is the number of band of the sensor, and N denotes the number of pixels per band.

**Figure 1:**
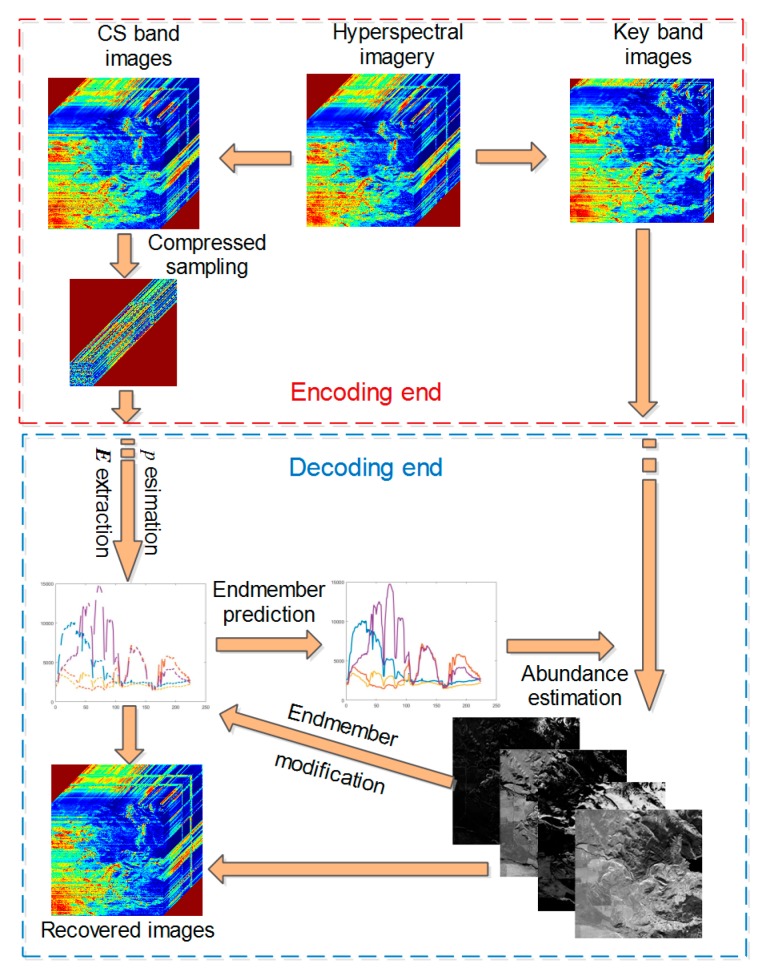
Framework of distributed compressed hyperspectral sensing (DCHS).

Figure 1 schematizes the proposed distributed compressed sampling strategy and reconstruction framework of CS band images. The DCHS framework consists of two parts: encoding end and decoding end.

For the distributed compressed sampling of the encoding end in Figure 1, we divided hyperspectral imagery into key band images and CS band images, represented by $X_{K}\in {\mathbb{R}}^{N\times L_{K}}$ and $X_{CS}\in {\mathbb{R}}^{N\times L_{CS}}$, respectively, where, $L_{K}$ is the number of key band and $L_{CS}=L-L_{K}$ is the number of the CS band.

First, hyperspectral imagery should be grouped in equal parts according to band, similar to the Group-of-Pictures (GOP) structure in many video codecs. $L_{g}$ denotes the number of bands in each group. The intermediate bands are then extracted from each group as the key band of the group. The key bands are transmitted directly to the decoding end without compressed sampling. This means that the equivalent sampling rate of the key band $SR_{K}$ is $L_{K}/L$. Band selection [29,30,31,32,33,34], according to hyperspectral feature, will provide a benefit to the performance of grouping. However, it will violate the requirement of the lowest computational cost at the encoding end of CS.

The remaining bands are taken as CS band images and the measurement matrices is defined as $A\in {\mathbb{R}}^{M\times N}$ (M≪N). Matrix A acts on the CS band along the spatial domain generating M measurements per band. The measurements obtained with matrix A are $Y_{CS}=AX_{CS}$. In our previous work [27], we designed a spatial measurement matrix, where each row is a one-hot vector. The designed matrix is still used in this paper for the spatial observation of CS band. The sampling rate of CS band $SR_{CS}$ is $ML_{CS}/NL$. As a consequence, the total equivalent sampling rate of DCHS is $SR=SR_{K}+SR_{CS}=(NL_{K}+ML_{CS})/NL$.

In hyperspectral imagery processing, LMM is an important and widely used model. LMM of hyperspectral imagery can be described in the following Equation.

(1) X=SE

For the key band images, Equation (1) can be rewritten as

(2) $$X_{K}=SE_{K}$$

where $E_{K}\in {\mathbb{R}}^{p\times L_{K}}$ denotes an endmember matrix of the key band holding the spectral signatures of the endmembers; $S\in {\mathbb{R}}^{N\times p}$ is the corresponding abundance matrix of key band, which describes the proportion fractions of ground materials at each pixel; and p is the number of endmember. As the CS band, the key band, and the original hyperspectral data describe the same scene and ground objects, the three data should have the same abundance matrix, S. Now, the compressed measurement of CS band can be written as

(3) $$Y_{CS}=AX_{CS}=ASE_{CS}$$

According to the proposed distributed compressed sampling mode, the task of DCHS reconstruction is to recover the CS band images, $X_{CS}$, as the key band is transmitted directly to the decoder. From Equation (3), we can see that the task of reconstructing $X_{CS}$ can be converted to the estimation of the endmember matrix $E_{CS}$ and the abundance matrix, S. Therefore, at the decoding end of Figure 1, the observed data of CS band images is first used to extract endmembers of the CS band. Then, the endmembers of the key band are predicted by these extracted endmembers. Afterwards, the abundance matrix can be estimated by combining the images and endmembers of the key band. Next, the endmember of CS bands is modified by the estimated abundance. Finally, the CS band images are reconstructed using the modified endmember and the estimated abundance fraction based on LMM.

## 3. Reconstruction Algorithm of CS Band

In this section, we focus on the reconstruction algorithm of DCHS for CS band images, which mainly includes endmember extraction and abundance estimation, where the endmember extraction includes the extraction of $E_{CS}$ from spatial compressed data and the prediction of $E_{K}$ by the extracted endmember matrix $E_{CS}$.

### 3.1. Endmember Extraction

The first goal is to extract $E_{CS}$ from $Y_{CS}$. Thanks to the designed measurement matrix A, the existing endmember extraction algorithms are suitable for the compressed data [27]. Vertex component analysis (VCA) [35] is one of the most popular endmember extraction algorithms for hyperspectral unmixing. In this paper, we employ the VCA algorithm to extract the endmember matrix $E_{CS}$ from $Y_{CS}$. We use Equation (4) for the endmember extraction,

(4) $$E_{CS}=vca(Y_{CS})$$

where vca denotes VCA endmember extraction algorithm.

Note that p will play an important role for the VCA algorithm. In the absence of noise, the rank of observed data matrix $Y_{CS}$ is precisely p. Some state-of-the-art subspace clustering algorithms [36,37,38,39,40] will help to accurately estimate the number of endmembers. However, the goal of CS is to reconstruct the original data rather than unmixing. In the experiments, we find that a p slightly higher than the real number of endmember can slightly improve the reconstruction accuracy. Hyperspectral signal identification by minimum error (HySime) [41] can estimate higher endmembers in most cases. Therefore, p is estimated from $Y_{CS}$ by the HySime algorithm.

Next, we must successfully predict $E_{K}$ before estimating abundance, although the abundance can also be estimated from $Y_{CS}$. However, due to the extremely low spatial sampling rate, accurate estimation of abundance is very difficult. Therefore, we turn to the prediction of $E_{K}$ to estimate S. We find that the matrix $E_{K}$ is composed of the column vectors extracted by interval $L_{g}$ from matrix E. The remaining column vectors composes the matrix $E_{CS}$. The interpolation method can locate the nearest data value, and assign the value according to the nearest data. Therefore, a simplest interpolation algorithm is employed to predict $E_{K}$ from the extracted endmember matrix $E_{CS}$ of CS band.

(5) $$E_{K}=interp(E_{CS})$$

where interp denotes interpolation method.

**Figure 2:**
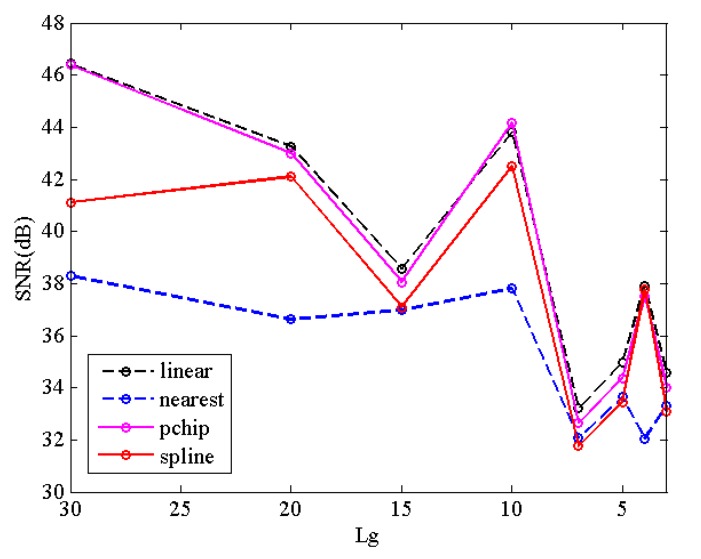
The performance of different interpolation methods.

Figure 2 evaluates the performance of several interpolation methods by average signal-to-noise ratio of (SNR) between the reference value and its estimated value predicted from the CS band. The spectral curves used for the evaluation come from the USGS library [42], which includes 501 spectral curves of different mineral types with 224 spectral bands. A total of 188 spectral bands remain after removing the water absorption and noise bands. $E_{K}$ is selected as reference value from the USGS library according to the grouping rules of the DCHS framework. Linear, nearest neighbor, spline, and shape-preserving piecewise cubic (pchip) interpolation methods are tested.

From Figure 2, we can see that linear and pchip methods achieve better prediction results than the other two interpolation methods. Linear interpolation is slightly better than pchip interpolation. Therefore, linear interpolation is used in all the following experiments.

### 3.2. Abundant Estimation

The next goal is to estimate the abundance matrix S after $E_{K}$ is successfully predicted. As described in the previous section, it is difficult to estimate abundance directly from $Y_{CS}$ due to the extremely low spatial sampling rate. Therefore, in this section, we combine $Y_{CS}$ and $X_{K}$ to estimate abundance. At the same time, the abundance characterizes the distribution map of different materials in the scene, which is a sparse signal on the orthogonal basis. Although sparse coding and feature representation-based methods [36,37,38,39,40] can better describe the sparsity of abundance, their use will significantly increase the computational complexity and contribute little to the final CS band reconstruction. This is because the modification of $E_{CS}$ in the next section can make up for the deficiency of abundance estimation. Therefore, we employ wavelet base as the orthogonal sparse basis.

Now, the abundant estimation task can be described as solving S, given observed data $Y_{CS}$, measurement matrix A, key band images $X_{K}$, and endmember matrix $E_{K}$ and $E_{CS}$. We consider the following constrained optimization problem,

(6) $$\underset{S}{\min}{\left\|WS\right\|}_{1,1}\;\mathrm{subject}\;\mathrm{to}\;X_{K}=SE_{K},\;Y_{CS}=ASE_{CS}$$

where ${\left\|C\right\|}_{1,1}\equiv {\sum_{i=1}^{p}\left\|c_{i}\right\|}_{1}$($c_{i}$ denotes the *i*th column of C, ${\left\|\cdot \right\|}_{1}$ denotes $\ell_{1}$ norm), and W represents an orthogonal wavelet base.

As the problem (6) is a non-convex optimization, we specialize the Alternating Direction Method of Multipliers (ADMM) [43,44] to optimize problem (6). First, by introducing regularization parameters, an equivalent way of writing the optimization problem (6) is the following unconstrained optimization problem,

(7) $$\underset{S}{\min}{\left\|WS\right\|}_{1,1}+\frac{\lambda_{1}}{2}{\left\|X_{K}-SE_{K}\right\|}_{F}^{2}+\frac{\lambda_{2}}{2}{\left\|Y_{CS}-ASE_{CS}\right\|}_{F}^{2}\;$$

where parameters $\lambda_{1}\geq 0$ and $\lambda_{2}\geq 0$ control the relative weight of the second and third terms in problem (7), respectively, and ${\left\|C\right\|}_{F}\equiv \sqrt{\mathrm{trace}\left\{CC^{T}\right\}}$ denotes the Frobenius norm of C. We introduce an auxiliary matrix Z=WS. Problem (7) can be written as

(8) $$\underset{Z}{\min}{\left\|Z\right\|}_{1,1}+\frac{\lambda_{1}}{2}{\left\|X_{K}-W^{-1}ZE_{K}\right\|}_{F}^{2}+\frac{\lambda_{2}}{2}{\left\|Y_{CS}-AW^{-1}ZE_{CS}\right\|}_{F}^{2}\;$$

where $W^{-1}$ is the inverse of matrix W.

Before the alternating minimization is apply to the corresponding augmented Lagrangian functions, we write the following equivalent formulation with auxiliary matrix $R_{1}$, $R_{2}$, and $R_{3}$,

(9) $$\begin{array}{l} \underset{Z,R_{1},R_{2},R_{3}}{\min}{\left\|Z\right\|}_{1,1}+\frac{\lambda_{1}}{2}{\left\|X_{K}-R_{2}E_{K}\right\|}_{F}^{2}+\frac{\lambda_{2}}{2}{\left\|Y_{CS}-AR_{3}\right\|}_{F}^{2}\\ \mathrm{subject}\;\mathrm{to}\;R_{1}=Z\\ \;R_{2}=W^{-1}R_{1}\\ \;R_{3}=R_{2}E_{CS} \end{array}$$

Constrained optimization problem (9) has an augmented Lagrangian subproblem of the form

(10) $$\begin{array}{l} \underset{Z,R_{1},R_{2},R_{3},T_{1},T_{2},T_{3}}{\min}{\left\|Z\right\|}_{1,1}+\frac{\lambda_{1}}{2}{\left\|X_{K}-R_{2}E_{K}\right\|}_{F}^{2}+\frac{\lambda_{2}}{2}{\left\|Y_{CS}-AR_{3}\right\|}_{F}^{2}\\ +\frac{\mu }{2}{\left\|Z-R_{1}-T_{1}\right\|}_{F}^{2}+\frac{\mu }{2}{\left\|W^{-1}R_{1}-R_{2}-T_{2}\right\|}_{F}^{2}+\frac{\mu }{2}{\left\|R_{2}E_{CS}-R_{3}-T_{3}\right\|}_{F}^{2} \end{array}$$

where μ>0 is a positive penalty constant; $T_{1}$, $T_{2}$, and $T_{3}$ denote the Lagrange multipliers.

For each iteration of ADMM, we first fix $R_{1},R_{2},R_{3}$ and $T_{1},T_{2},T_{3}$; the minimizer of objective function (10) with respect to Z is the well-known soft threshold problem [45], and the problem can be reduced to

(11) $$\underset{Z}{\min}{\left\|Z\right\|}_{1,1}+\frac{\mu }{2}{\left\|Z-R_{1}^{k}-T_{1}^{k}\right\|}_{F}^{2}$$

The soft threshold to solve problem (11) is given by

(12) $$Z^{k+1}=soft\left(R_{1}^{k}+T_{1}^{k},\frac{1}{\mu }\right)$$

Next, given other variables, simple manipulation shows that the minimization of objective function (10) with respect to $R_{1}$ is equivalent to

(13) $$\underset{R_{1}}{\min}{\left\|Z^{k+1}-R_{1}-T_{1}^{k}\right\|}_{F}^{2}+{\left\|W^{-1}R_{1}-R_{2}^{k}-T_{2}^{k}\right\|}_{F}^{2}$$

which is a least squares problem, and the corresponding normal Equation is

(14) $$\left(I_{N}+WW^{-1}\right)R_{1}=Z^{k+1}-T_{1}^{k}+W\left(R_{2}^{k}+T_{2}^{k}\right)$$

where $I_{N}$ denotes the N×N identity matrix. As W is the orthonormal basis, $WW^{-1}=I_{N}$. Therefore, the solution $R_{1}^{k}$ of Equation (14) is given easily by

(15) $$R_{1}^{k+1}=\frac{1}{2}\left[Z^{k+1}-T_{1}^{k}+W\left(R_{2}^{k}+T_{2}^{k}\right)\right]$$

Similarly, the steps to compute the values of the variables $R_{2}$ and $R_{3}$ are also least squares problems. The value of $R_{2}$ is given by

(16) $$R_{2}^{k+1}=\left[\lambda_{1}X_{K}E_{K}^{T}+\mu W^{-1}R_{1}^{k+1}-\mu T_{2}^{k}+\mu \left(R_{3}^{k}+T_{3}^{k}\right)E_{CS}^{T}\right]{\left(\lambda_{1}E_{K}E_{K}^{T}+\mu I_{p}+\mu E_{CS}E_{CS}^{T}\right)}^{-1}$$

where $E_{K}^{T}$ is the transpose of matrix $E_{K}$, and $I_{p}$ is a p×p identity matrix. The value of $R_{3}$ is given by

(17) $$R_{3}^{k+1}={\left(\lambda_{2}A^{T}A+\mu I_{N}\right)}^{-1}\left[\lambda_{2}A^{T}Y_{CS}+\mu \left(R_{2}^{k+1}E_{CS}-T_{3}^{k}\right)\right]$$

As the number of pixels N is usually large, ${\left(\lambda_{2}A^{T}A+\mu I_{N}\right)}^{-1}$ often requires enormous computation time. However, the designed measure measurement matrices A and $I_{N}$ are sparse matrices. The inverse operation is easy to perform. Moreover, the inversion only needs to be calculated once, as $\lambda_{2}A^{T}A+\mu I_{N}$ is unchanged for each iteration.

Finally, we update Lagrange multipliers by

(18) $$\begin{array}{l} T_{1}^{k+1}=T_{1}^{k}-\left(Z^{k+1}-R_{1}^{k+1}\right)\\ T_{2}^{k+1}=T_{2}^{k}-\left(W^{-1}R_{1}^{k+1}-R_{2}^{k+1}\right)\\ T_{3}^{k+1}=T_{3}^{k}-\left(R_{2}^{k+1}E_{CS}-R_{3}^{k+1}\right) \end{array}$$

After the kth iteration, the residual is defined as

(19) $$\begin{array}{l} res1={{\left\|X_{K}-S^{k+1}E_{K}\right\|}_{F}/\left\|X_{K}\right\|}_{F}\\ res2={{\left\|Y_{CS}-AS^{k+1}E_{CS}\right\|}_{F}/\left\|Y_{CS}\right\|}_{F} \end{array}$$

The iteration stopping criterion is defined as $res1<\varepsilon_{1}$ and $res2<\varepsilon_{2}$.

### 3.3. Recovery of CS Band

Although we have extracted the endmember $E_{CS}$ by VCA algorithm in Section 3.1 and estimated the abundance S in Section 3.2, the endmember and abundance are not directly matched, and this may reduce the reconstruction accuracy. Therefore, we modify $E_{CS}$ to minimize the objective function ${\left\|Y_{CS}-AS^{k+1}E_{CS}\right\|}_{F}^{2}$, whose least squares solution is given

(20) $${\hat{E}}_{CS}={\left({\left(AS^{k+1}\right)}^{T}AS^{k+1}\right)}^{-1}{\left(AS^{k+1}\right)}^{T}Y_{CS}$$

Finally, the CS band can be reconstructed by the LMM.

(21) $${\hat{X}}_{CS}=S^{k+1}{\hat{E}}_{CS}$$

In summary, we call the proposed CS band reconstruction algorithm a DCHS reconstruction algorithm, which is described in Algorithm 1.
**Algorithm 1:** DCHS reconstruction algorithm **Inputs:**$X_{K}$, $Y_{CS}$, and A **Output:**${\hat{X}}_{CS}$ 1. Estimate p by HySime algorithm from $Y_{CS}$ 2. Extract $E_{CS}$ from $Y_{CS}$ by VCA algorithm 3. Predict $E_{K}$ by $E_{CS}$ using interpolation algorithm 4. Set parameters: $\lambda_{1}$, $\lambda_{2}$, μ and maxiters 5. Initialize: $S^{0}=X_{K}E_{K}^{T}{\left(E_{K}E_{K}^{T}\right)}^{-1}$, $Z^{0}=WS^{0}$, $R_{1}^{0}=Z^{0}$, $R_{2}^{0}=W^{-1}R_{1}^{0}$, $R_{3}^{0}=R_{2}^{0}E_{CS}$, $T_{1}^{0}=0$, $T_{2}^{0}=0$, $T_{3}^{0}=0$, k=1, $thr=10^{-5}$, res1=res2=∞ 6. While k<maxiters and (res1>thr or res2>thr) 7. Compute $Z^{k+1}$ by soft-threshold function according to (12) 8. Compute $R_{1}^{k+1}$ by (15)  9. Compute $R_{2}^{k+1}$ by (16)  10. Compute $R_{3}^{k+1}$ by (17) 11. Update Lagrange multipliers $T_{1}^{k+1}$, $T_{2}^{k+1}$, and $T_{3}^{k+1}$ by (18) 12. Compute res1 and res2 by (19) 13. $S^{k+1}=W^{-1}Z^{k+1}$, k=k+1 End while 14. Modify $E_{CS}$ by (20) 15. Recover CS band according to LMM by (21)

In the DCHS reconstruction algorithm, the computational complexity is mainly reflected in the estimation of the abundance due to multiple iterations. In each iteration of abundance estimation, the most costly steps are the calculus of $R_{2}^{k+1}$ and $R_{3}^{k+1}$, both of which have the order of complexity $O(pNL_{CS})$, where p is the number of endmembers, N is the number of pixels in the image, and $L_{CS}$ is the number of CS spectral bands.

## 4. Experiments and Results

In this section, we compare the proposed DCHS framework with several state-of-the-art reconstruction algorithms to evaluate the validity of the proposed framework, including MT-BCS [46], CPPCA [10], SSHCS [27], SpeCA [15], and SSCR_SU [28]. In the comparison experiments, we used the default parameter settings of those compared methods described in the reference papers. It is worth noting that the SpeCA algorithm cannot estimate the number of endmember. Therefore, in comparison experiments, we set it according to the ground truth. All the experiments were run with MATLAB 2014a (32-bit) on a laptop workstation with 2.6 GHz CPU and 32 GB RAM. We quantitatively and visually evaluated the performance of the proposed method on three real datasets, namely, Cuprite and Urban from the hyperspectral unmixing datasets [47], and PaviaU from hyperspectral remote sensing scenes [48].

The Cuprite dataset contains 188 bands via removing water absorption and noise bands, including 250 × 190 pixels. In general, the Cuprite dataset is considered to contain 14 types of minerals. The Urban dataset is of size 306 × 306 and consists of 162 bands. There are six endmembers contained in the ground truth. PaviaU dataset is of 610 × 340 pixels and 103 bands. The ground truths differentiate nine classes. The false-color images of the three dataset are shown in Figure 6a. The red, green, and blue channels are (40,20,10) bands for Cuprite dataset, (28,11,2) bands for Urban dataset, and (50,30,5) bands for PaviaU dataset, respectively.

In order to evaluate the reconstruction performances of all methods, three quantitative indices are employed in the experiments. The first index is mean peak signal-to-noise ratio (MPSNR) between the reconstructed images and the original images, which is defined as the average peak signal-to-noise ratio (PSNR) of all bands. MPSNR is defined as

(22) $$\mathrm{MPSNR}=\frac{1}{L}\sum_{i=1}^{L}20\log_{10}\frac{\max(X_{i})}{\sqrt{{\left\|X_{i}-{\hat{X}}_{i}\right\|}_{2}^{2}/N}}$$

where $X_{i}$ and ${\hat{X}}_{i}$ correspond to the original and reconstructed band image vector. $\max(X_{i})$ is the peak value of $X_{i}$. High values of MPSNR represent better reconstruction results.

The second index is mean spectral angle mapper (MSAM), which calculates the average angle between the original and reconstructed spectral vectors for all spatial positions; its definition is as follows,

(23) $$\mathrm{MSAM}=\frac{1}{N}\sum_{j=1}^{N}\arccos\frac{X_{j}^{T}\cdot {\hat{X}}_{j}}{{\left\|X_{j}\right\|}_{2}\cdot {\left\|{\hat{X}}_{j}\right\|}_{2}}$$

where $X_{j}$ and ${\hat{X}}_{j}$ are the *j*th spectral vectors of the original and reconstructed HSI, respectively. Low values of MSAM represent better reconstruction results.

The last index, mean structure similarity (MSSIM), is used to evaluate the structural consistency between the original and reconstructed HSI, which is expressed as

(24) $$\mathrm{MSSIM}=\frac{1}{L}\sum_{i=1}^{L}\mathrm{SSIM}(X_{i},{\hat{X}}_{i})$$

where $\mathrm{SSIM}(X_{i},{\hat{X}}_{i})$ is defined as the structure similarity of between $X_{i}$ and ${\hat{X}}_{i}$. For the details of the SSIM function the reader can refer to work in [49].

**Figure 3:**
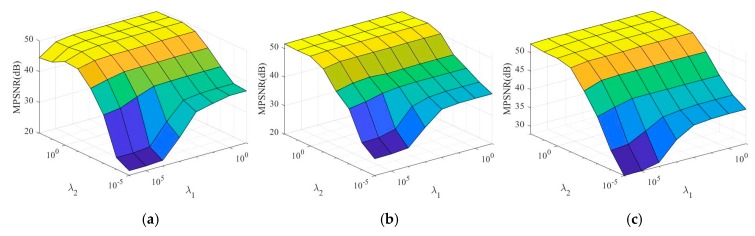
Sensitivity analysis of the regularization parameters $\lambda_{1}$ and $\lambda_{2}$ of the proposed DCHS algorithm with different number of bands in each group $L_{g}$ or different sampling rates SR. (**a**) $L_{g}=20$, SR=0.0564, (**b**) $L_{g}=10$, SR=0.1048, and (**c**) $L_{g}=5$, SR=0.2048.

The first group experiments discuss the parameter setting of the proposed algorithm by Cuprite dataset. In our DCHS reconstruction algorithm, there are three important parameters: $\lambda_{1}$, $\lambda_{2}$, and μ. First, we fix parameter μ=30, and set the number of bands in each group $L_{g}=20,10,5$, corresponding to 0.0564, 0.1048, and 0.2048 sampling rate. Figure 3 shows the trends of MPSNR with $\lambda_{1}$ and $\lambda_{2}$.

From Figure 3, we can see that the change trends of MPSNR with $\lambda_{1}$ and $\lambda_{2}$ are basically the same for different $L_{g}$. Therefore, for different sampling rates, we can use the same setting for parameters $\lambda_{1}$ and $\lambda_{2}$. In addition, MPSNR changes significantly more along the $\lambda_{2}$-axis direction than along the $\lambda_{1}$-axis direction, which means that the proposed DCHS reconstruction algorithm is more sensitive to $\lambda_{2}$. MPSNR increases when $\lambda_{2}$ increases. When $\lambda_{2}$ is greater than 1, MPSNR increases very little. Therefore, in the following experiments, we set the parameters $\lambda_{1}=10^{4}$, $\lambda_{2}=1$.

**Figure 4:**
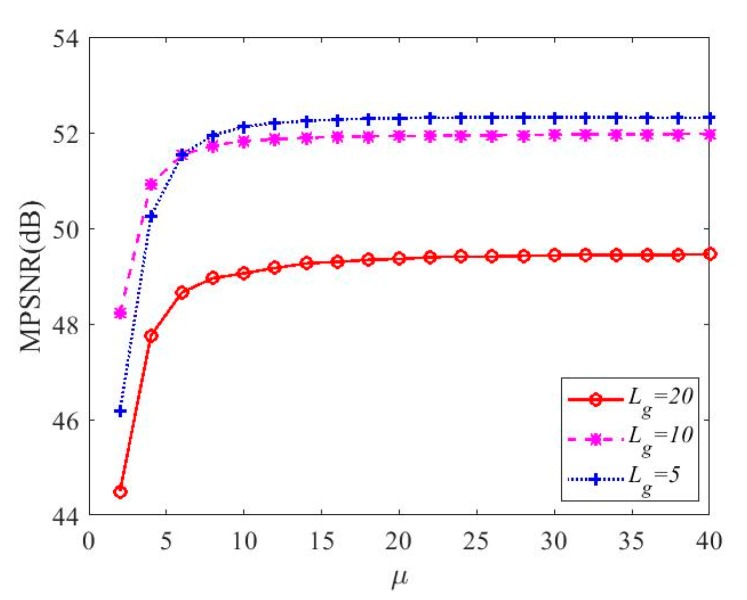
Sensitivity analysis of the parameter μ of the proposed DCHS algorithm with different number of bands in each group $L_{g}$.

Figure 4 shows the influence of parameter μ on the reconstruction performance with different sampling rates. We can see that as μ increases, the reconstructed MPSNR gradually increases. When μ is less than 10, the MPSNR increase rapidly. When μ exceeds 30, the MPSNR is basically unchanged. Therefore, in our following experiments, we set parameter μ=30.

The second group experiments compare the reconstruction performance of the proposed approach with respect to the state-of-the-art methods for the above three datasets. In these experiments, we give the changes of MPSNR, MSAM, and MSSIM of several reconstruction algorithms with the sampling rate. Because the sampling rate is a consistent indicator of compressed sensing references, although some references describe other forms of sampling rates, such as the number of bands per group $L_{g}$, in this paper, they can be converted to sampling rates indicator. As the sampling rate of the proposed DCHS depends on $L_{g}$, we test DCHS using different values of $L_{g}$: 30, 20, 15, 10, 7, 5, 4, 3, corresponding to different sampling rates. For example, for the Cuprite dataset, the corresponding sampling rates are 0.0416, 0.0564, 0.0732, 0.1048, 0.1469, 0.2048, 0.2575, and 0.3365; for the Urban dataset, the corresponding sampling rates are 0.0406, 0.0589, 0.0711, 0.1078, 0.1506, 0.2056, 0.2544, and 0.34; for the PaviaU dataset, the corresponding sampling rates are 0.0388, 0.0581, 0.0677, 0.1061, 0.1446, 0.2022, 0.2503, and 0.3368.

**Figure 5:**
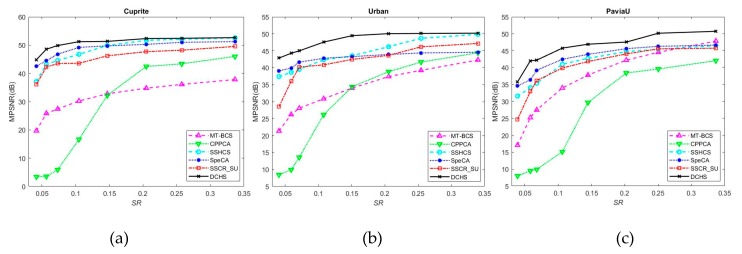
Mean peak signal-to-noise ratio (MPSNR) curves of different algorithms for different datasets: (**a**) Cuprite dataset, (**b**) Urban dataset, and (**c**) PaviaU dataset.

Figure 5 shows the comparison results of MPSNR of different algorithms for different datasets. For the Cuprite dataset, the proposed DCHS algorithm shows its superiority at low sampling rates. For example, around 0.05 sampling rate, it is more than 4dB higher than the SpeCA algorithm with the best performance. However, this advantage gradually diminishes as the sampling rate increases. When the sampling rate exceeds 0.25, the DCHS algorithm achieves almost the same MPSNR values as the SSHCS. This is because when the sampling rate is increased, the value of $L_{g}$ will become smaller, and the endmember prediction accuracy of key band images will reduce, thereby affecting the reconstruction performance. In addition, it can be seen from the Figure 5a that CPPCA algorithm fails at low sampling rate. When the sampling rate exceeded 0.15, the reconstruction performance of CPPCA exceeds that of MT-BCS, and SSHCS exceeds that of SpeCA. Although the performance of CPPCA improves rapidly with the increase of sampling rate, it still lags behind other LMM-based reconstruction algorithms. For example, the MPSNR of DCHS is more than 5 dB higher than CPPCA with a higher sampling rate. The results further prove that hyperspectral compressed sensing reconstruction based on LMM, such as DCHS, SSCR_SU, SpeCA, and SSHCS, is better than the reconstruction algorithms without using LMM, such as CPPCA and MT-BCS.

Similar to the results of the Cuprite dataset, DCHS can still obtain the best reconstruction performance for the Urban and PaviaU datasets. It is worth mentioning that, unlike the experiment results of Cuprite and Urban, the reconstruction performance of the DCHS on the PaviaU dataset is also excellent even at a high sampling rate. It further illustrates the effectiveness of the proposed DCHS framework. In addition, the MT-BCS algorithm also performs very well on the PaviaU dataset. When the sampling rate exceeds 0.3, the MT-BCS algorithm is superior to other reconstruction algorithms except DCHS.

**Figure 6:**
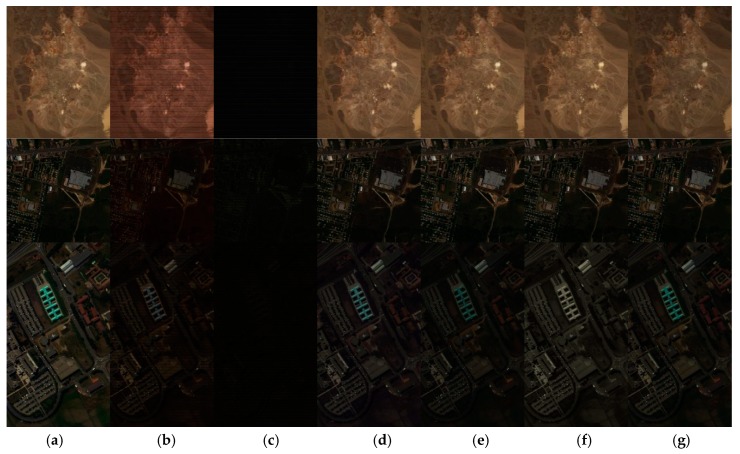
Original and reconstructed pseudocolor images achieved by different algorithms on different datasets near the 0.05 sampling rate, from top to bottom Cupriete, Urban, and PaviaU: (**a**) original, (**b**) MT-BCS, (**c**) CPPCA, (**d**) SSHCS, (**e**) SpeCA, (**f**) SSCR_SU, and (**g**) DCHS.

**Figure 7:**
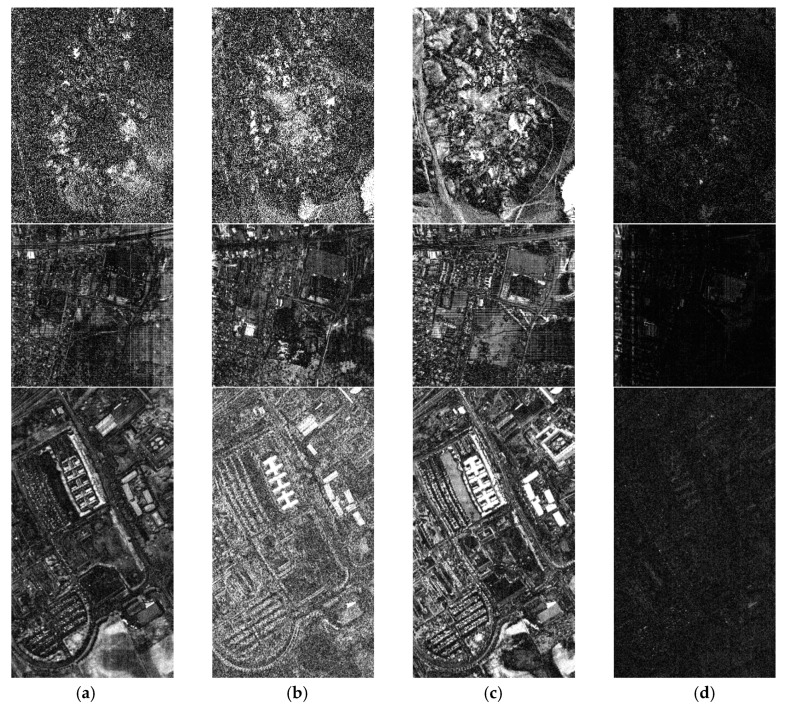
The 28th band residual images of different algorithms on different datasets near the 0.05 sampling rate, from top to bottom Cupriete, Urban, and PaviaU: (**a**) SSHCS, (**b**) SpeCA, (**c**) SSCR_SU, (**d**) DCHS.

Figure 6 shows the visual qualities of the original and reconstructed pseudocolor images for the different datasets. The sampling rate is set to 0.0564, 0.0589, and 0.0581 for Cuprite, Urban, and PaviaU dataset, respectively. It can be seen from the figure that the CPPCA algorithm can hardly recover the original image near the 0.05 sampling rate. The reconstruction quality of MT-BCS is also very poor. The compressed sensing reconstruction algorithms based on LMM can recover the original image better, and the spatial details are well preserved. However, slight color distortion can still be observed on the PaviaU dataset. This color distortion phenomenon indicates that the LMM-based reconstruction algorithm has excellent performance in preserving spatial information, but is poor in maintaining spectral information. The advantages and disadvantages of SSHCS, SpeCA, SSCR_SU, and DCHS algorithms are hard to distinguish visually. Actually, they have subtle color distortions that make it difficult for the human eye to distinguish. Therefore, in order to illuminate the visual difference of reconstructed images achieved by LMM-based algorithms, we demonstrate the residual images between the original images and the reconstructed images in Figure 7.

Figure 7 shows the residual images at the 28th band of the three datasets. Note that the residuals of each dataset of all algorithms are amplified at the same scale. The brighter the residual image, the larger the residual, that is, the worse the reconstruction performance of the algorithm. The results of Figure 7 clearly demonstrate the effectiveness of the proposed DCHS algorithm. No matter which dataset, the residual images achieved by DCHS are obviously darker than the other three LMM-based algorithms. For the Cuprite and Urban datasets, the residual images of SSCR_SU brighter than that of SSHCS and SpeCA. The residual image of SpeCA is brightest on PaviaU dataset.

Table 1 illuminates the MSAM by various reconstruction algorithms. It can be seen from the experimental results that the proposed DCHS algorithm does not always lead with the spectral angle mapper, which may be caused by the low prediction accuracy of the endmember of key band by DCHS. A more accurate prediction algorithm may help reduce MSAM, but this is beyond the scope of the article. Even so, for the Urban dataset, the MSAM at all sampling rates is lower than other algorithms. For other two datasets, DCHS is still very close to the optimal result. For some specific sampling rates, DCHS still outperforms the other algorithms.

**Figure 8:**
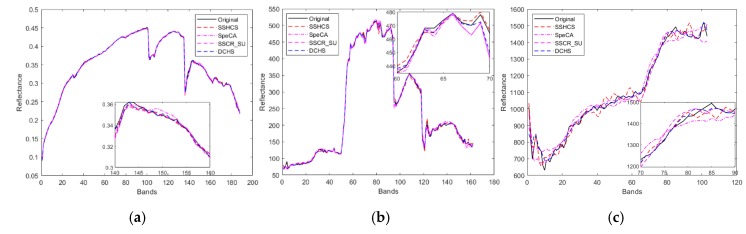
Spectral curves of original and reconstructed achieved by different algorithms. (**a**) Cuprite dataset, (**b**)Urban dataset, (**c**) PaviaU dataset.

The comparison of the original and reconstructed spectral curves is shown in Figure 8. The sampling rates of Cuprite, Urban, and PaviaU are 0.3365, 0.34, and 0.3368, respectively. We also provide locally enlarged subgraphs. As the spectral deviation of the MT-BCS and CPPCA algorithms is serious, these two algorithms are removed from Figure 8 in order to clearly show the contrast effect. As can be seen from the figure, Cuprite has the highest spectral matching for all algorithm, while PaviaU has the worst, which is also consistent with MSAM in Table 1. It is possible that the reconstruction algorithms based on LMM are sensitive to the number of bands; the higher the number of bands, the better the reconstruction performance.

From locally enlarged subgraphs in Figure 8, the SpeCA algorithm for the Cuprite dataset is the worst, and the SSCR_CU algorithm for the Urban dataset is the worst. However, the spectral curves recovered by several algorithms for PaviaU dataset are poor. DCHS and SSHCS are closer to the original spectral curves. However, this is only a local feature and cannot explain the advantages and disadvantages of each algorithm. To evaluate the reconstruction algorithm on spectral domain, it is also necessary to refer to the statistical indicators of all pixels, such as MSAM.

The experimental results of MSSIM are shown in Table 2, which is similar to Table 1. In most cases, the proposed DCHS can achieve the highest MSSIM value; although it is not optimal in a few cases, it is still close to optimal. MT-BCS and CPPCA performed worst in both Table 1 and Table 2. The effectiveness of the LMM-based hyperspectral compressed sensing reconstruction algorithm is further confirmed.

In the last experiment, the runtime is measured in order to compare the computational complexity of algorithms. Herein, we use the Cuprite dataset to evaluate the speed of the algorithms. Table 3 presents the runtimes of different algorithms on Cuprite dataset. The running time of CPPCA and SSHCS is on the same order of magnitude, achieving the fastest reconstruction speed. The computational complexity of MT-BCS, SpeCA, and DCHS is equivalent, and the running time is in the same order of magnitude.

## 5. Conclusions

In this paper, inspired by DCVS, we proposed a compressed sensing framework for hyperspectral imagery, called DCHS, which first decomposes hyperspectral data into the CS band and key band for compressed sampling. To effectively recover original hyperspectral imagery from compressed data based on the proposed compressed sampling framework, we discarded side information based reconstruction of DCVS and developed a hyperspectral reconstruction algorithm based on spectral unmixing for distributed compressed sampling. The reconstruction process is converted to the estimation of the endmember and its corresponding abundance fraction. A method combining endmember extraction and prediction was proposed for key band endmembers estimation. The optimization algorithm of joint abundance sparsity, key and CS band observation data fidelity was also designed for abundance estimation. By analyzing the experimental results on three real datasets, we found that the proposed framework is beneficial to reconstruct the original data by LMM. More notably, the proposed method is able to obtain a more accurate peak signal-to-noise ratio compared to other state-of-the-art reconstruction algorithms.

However, the proposed DCHS cannot always lead the recovery of the spectral curve. Therefore, in future work, we will look for accurate endmember prediction algorithms in order to recover the spectral curve with high precision.

## Figures and Tables

**Table 1:** Comparison of mean spectral angle mapper (MSAM) (°) achieved by the various algorithms (the best results are in bold).

$L_{g}$	30	20	15	10	7	5	4	3
**Results on the Cuprite Dataset**								
SR	0.0416	0.0564	0.0732	0.1048	0.1469	0.2048	0.2575	0.3365
MT-BCS	13.135	6.5641	5.6391	4.4379	3.5327	3.0821	2.8048	2.529
CPPCA	87.408	82.683	63.714	21.502	3.5086	0.9931	0.9064	0.6554
SSHCS	1.8222	0.9963	0.8775	1.1346	0.6602	**0.4074**	**0.387**	**0.3658**
SpeCA	**0.8355**	0.8227	**0.6226**	**0.5005**	**0.4706**	0.4523	0.4222	0.4079
SSCR_SU	1.7216	1.0615	0.983	0.9051	0.7575	0.6331	0.5846	0.5059
DCHS	0.9694	**0.7088**	0.6528	0.5735	0.5083	0.4901	0.4537	0.4352
**Results on the Urban Dataset**								
SR	0.0406	0.0589	0.0711	0.1078	0.1506	0.2056	0.2544	0.34
MT-BCS	20.949	12.691	10.356	7.7535	5.5784	3.9322	3.1864	2.241
CPPCA	87.626	73.818	50.011	13.114	4.9118	3.1499	2.3014	1.7288
SSHCS	3.8007	3.5748	3.1717	2.2219	2.0846	1.4805	1.1498	1.0011
SpeCA	3.2096	2.8107	2.5085	2.1831	2.0845	2.0381	1.9604	1.9545
SSCR_SU	8.9135	4.6612	2.94	2.6452	2.3153	2.1638	1.6217	1.3918
DCHS	**2.671**	**2.2361**	**2.0754**	**1.533**	**1.2448**	**1.1214**	**1.0631**	**0.9546**
**Results on the PaviaU Dataset**								
SR	0.0388	0.0581	0.0677	0.1061	0.1446	0.2022	0.2503	0.3368
MT-BCS	41.148	15.13	11.286	5.7488	3.6916	2.3748	1.8101	1.2232
CPPCA	88.814	87.689	82.943	59.258	8.9603	3.4445	3.1112	2.357
SSHCS	8.2997	6.0203	4.7841	2.8518	2.3971	1.8155	1.6073	1.4682
SpeCA	**4.9424**	4.1384	3.3207	**2.3286**	**2.0295**	**1.809**	1.6015	1.5467
SSCR_SU	15.2691	5.7259	5.0558	3.477	2.6279	2.144	1.7809	1.7833
DCHS	5.5415	**3.211**	**3.0072**	2.4134	2.2623	2.1816	**1.3542**	**1.1391**

**Table 2:** Comparison of MSSIM achieved by the various algorithms (the best results are in bold).

$L_{g}$	30	20	15	10	7	5	4	3
**Results on the Cuprite Dataset**								
SR	0.0416	0.0564	0.0732	0.1048	0.1469	0.2048	0.2575	0.3365
MT-BCS	0.2987	0.6297	0.7213	0.8332	0.91	0.9461	0.9604	0.9729
CPPCA	0.0001	0.0026	0.0191	0.3278	0.9535	0.9862	0.9876	0.9926
SSHCS	0.9624	0.987	0.9855	0.9916	0.9940	**0.9962**	**0.9965**	**0.997**
SpeCA	**0.9863**	0.9875	**0.9912**	**0.9946**	**0.9953**	0.9956	0.9961	0.9964
SSCR_SU	0.9737	0.988	0.9874	0.9876	0.9896	0.9925	0.9933	0.9949
DCHS	0.9857	**0.9888**	0.9902	0.9922	0.9938	0.994	0.9949	0.9953
**Results on the Urban Dataset**								
SR	0.0406	0.0589	0.0711	0.1078	0.1506	0.2056	0.2544	0.34
MT-BCS	0.4105	0.614	0.6823	0.7563	0.828	0.8924	0.9158	0.9487
CPPCA	0.0051	0.0332	0.2008	0.6924	0.8842	0.9393	0.9609	0.9734
SSHCS	0.9443	0.9424	0.9314	0.959	0.9558	0.973	0.9832	0.9863
SpeCA	0.9467	0.9474	0.9675	0.9741	0.9754	0.9793	0.9795	0.9804
SSCR_SU	0.8762	0.9344	0.9648	0.9711	0.9742	0.9771	0.9822	0.9839
DCHS	**0.9667**	**0.9722**	**0.9749**	**0.9825**	**0.9857**	**0.9871**	**0.9883**	**0.9901**
**Results on the PaviaU Dataset**								
SR	0.0388	0.0581	0.0677	0.1061	0.1446	0.2022	0.2503	0.3368
MT-BCS	0.124	0.4773	0.5716	0.7687	0.8617	0.9239	0.9492	0.9742
CPPCA	0.0068	0.0072	0.0173	0.1254	0.7075	0.9013	0.9154	0.9351
SSHCS	0.803	0.8714	0.8928	**0.9445**	**0.9585**	**0.9717**	**0.9755**	0.9814
SpeCA	0.8149	0.863	0.8841	0.9341	0.9471	0.958	0.9641	0.9655
SSCR_SU	0.6054	0.8653	0.8354	0.9105	0.9383	0.945	0.9566	0.9565
DCHS	**0.861**	**0.9187**	**0.92**	0.9413	0.9505	0.9572	**0.9755**	**0.9834**

**Table 3:** Comparison of runtime(s) for the various algorithms on Cuprite dataset.

$L_{g}$	30	20	15	10	7	5	4	3
SR	0.0416	0.0564	0.0732	0.1048	0.1469	0.2048	0.2575	0.3365
MT-BCS	19.0391	22.1187	17.7807	25.6069	29.3980	34.6212	43.4569	52.9737
CPPCA	0.1005	0.0627	0.0585	0.1006	0.1066	0.1727	0.2139	0.5978
SSHCS	0.2831	0.1351	0.1269	0.0917	0.1132	0.1012	0.0919	0.0932
SpeCA	15.5695	30.4764	49.2695	58.9999	58.9444	59.8885	57.1876	56.5255
SSCR_SU	4.2837	3.3935	1.2450	3.4530	1.3813	1.2545	1.3005	1.3809
DCHS	33.0788	34.6071	36.2856	34.2655	33.2441	30.8957	29.1711	26.9435
